# Diet diversity score and healthy eating index in relation to diet quality and socio-demographic factors: results from a cross-sectional national dietary survey of Swedish adolescents

**DOI:** 10.1017/S1368980019004671

**Published:** 2020-07

**Authors:** Lotta Moraeus, Anna Karin Lindroos, Eva Warensjö Lemming, Irene Mattisson

**Affiliations:** Department of Risk and Benefit Assessment, Swedish Food Agency, se-751 26 Uppsala, Sweden

**Keywords:** Socio-economic status, Dietary indices, Adolescents, Diet diversity, National dietary survey

## Abstract

**Objective:**

Groups with low socio-economic status have less healthy diets and higher prevalence of non-communicable diseases. Using the latest Swedish national dietary survey data, we developed a healthy eating index and a diet diversity score with the aim to explore associations between the scores and socio-demographic factors.

**Design:**

Cross-sectional national dietary survey. A web-based retrospective registration of food and beverages during 2 d was used to assess dietary intake. This information was used to construct the Swedish Healthy Eating Index for Adolescents 2015 (SHEIA15) and the Riksmaten Adolescents Diet Diversity Score (RADDS). The scores were based on the latest Swedish dietary guidelines from 2015. Intakes of food and nutrients across the scores were examined. Mixed-effects multilevel models were used to assess associations between the scores and household education, sex, school grade, weight status and school municipality.

**Setting:**

School-based survey in Sweden.

**Participants:**

2905 adolescents in grades 5, 8 and 11, 56 % girls.

**Results:**

High scores on SHEIA15 and RADDS were associated with higher intake of vegetables, fish and several nutrients, and lower intake of sugar-sweetened beverages and red meat. Boys and participants in households with lower education level scored lower on both indices. Individuals with overweight/obesity scored lower on RADDS.

**Conclusions:**

The newly developed indices can be used to identify healthy eating patterns among Swedish adolescents. Both indices show that boys and adolescents from households with lower education level have poorer dietary habits. Lower diet diversity was related to overweight/obesity, but the overall healthy eating index was not.

Dietary habits are related to several health outcomes such as heart disease, cancer and obesity^(1)^. Diets that are rich in vegetables, legumes, wholegrains, fish and oils are associated with lower risk of these health outcomes than diets with high intake of red meat, added sugar and salt^(2)^. According to estimations by Global Burden of Disease, poor dietary habits are one of the most important lifestyle contributors to the disease burden in Sweden as well as worldwide^(3)^. Adherence to a healthy diet is unevenly distributed in the population, and socio-demographic factors are associated with dietary habits. Lower socio-economic status is associated with less nutrient dense diets^(4)^ as well as with lower intakes and nutritional status of vitamins and minerals^(5)^. The associations have been documented in a number of studies using different indicators for socio-economic status and ways to describe dietary habits^(4–7)^. In a European setting, Alkervi *et al.* found that education level was associated with healthy food choices, while economic resources were associated with food diversity and energy density^(6)^. A review report from the Swedish Food Agency (SFA) shows that parental education, especially the education level of the mother, is positively associated with healthier food habits among children and that healthy food habits are associated with higher food costs^(8)^. In the latest national dietary survey in Sweden, Riksmaten Adolescents 2016–2017, adolescents in households with higher education level ate vegetables and fish more often and drank sugar-sweetened beverages less often than those from households with lower education level. Furthermore, the intake of several nutrients, such as vitamin D, iodine and Fe, was higher in the adolescents from households with high education^(9,10)^.

In Sweden as well as other European countries, prevalence of childhood obesity is higher in rural than in urban areas^(11,12)^. This difference can partly but not entirely be explained by other socio-economic factors such as parental education level^(11,12)^. Surveys on urban–rural differences in dietary habits show conflicting results. In a Finnish national survey, children living in semi-urban areas had poorer diet quality than those in urban areas, as measured by the Finnish Children Healthy Eating Index^(13)^. In contrast, a regional survey in South West Britain found healthier dietary patterns in rural children compared with urban, including lower intake of snacks and processed food^(14)^.

Dietary intake is a complex exposure, and there are many different options for describing diet intake and quality. They include intake of foods or nutrients, dietary patterns and biomarkers of nutrient status. Analyses using single components like nutrient intakes are often used but are problematic when trying to understand the impact of the overall diet, since nutrients or food items are not consumed in isolation. One way to overcome this is to create dietary patterns, which are tools to measure the multidimensional aspects of food intake. Dietary patterns can be evaluated with data-driven methods like principal component analysis or cluster analysis, or using different *a priori* methods^(15)^. Numerous diet indices to capture diet quality have been developed, but there is no consensus on choice of method^(16,17)^. Indices can measure diet quality as conformance to dietary guidelines such as the Healthy Eating Index^(18)^, while other indices capture special features of the diet such as the Mediterranean diet^(19)^ and dietary inflammatory index^(20)^. All these indices are predefined, using cut-offs based on current nutrition knowledge, and have been related to several health outcomes. Adherence to the Mediterranean diet has proven protective against CVD^(19)^ and similarly, high scores on the Healthy Eating Index have been associated with lower risk of all-cause mortality and CVD^(21)^. Another dimension of the diet is variation. Diet diversity scores are based on the number of food groups or the number of foods consumed. High diet diversity scores have been associated with better overall nutrient composition of the diet^(22,23)^. Furthermore, low-diet diversity score was associated with lower social class and economic hardship in a British study^(24)^.

Indices are developed to suit context and food culture of a country or larger geographical area. Healthy eating indices should ideally be updated when new knowledge and recommendations emerge^(18)^. There are scores evaluating aspects of the Nordic diet such as the Baltic Sea Diet Score^(25)^ and an index evaluating the Nordic nutrition recommendations in 2011^(26)^. However, there are no indices based on the Swedish food-based dietary guidelines from 2015 or diet diversity score to capture variety in the diet in Swedish adolescents.

The aims of the current study were: (a) to develop two different indices reflecting healthy eating and diet diversity, (b) to compare nutrient and food intakes across different levels of these indices and (c) to examine the associations between the respective indices and socio-demographic characteristics in the national cross-sectional dietary survey ‘Riksmaten Adolescents 2016–2017’ in Sweden.

## Methods

### Study design

‘Riksmaten Adolescents 2016–2017’ is a cross-sectional dietary survey conducted by the SFA. The survey included a national sample of adolescents in school grade 5, 8 and 11 in Sweden. Diet was assessed using a web-based method, RiksmatenFlex, and height and weight were measured. Participants also filled in questionnaires on background, food habits and intake of foods consumed less frequently. A detailed description of the study design, methods and participation is presented by Moraeus *et al.*^(27)^.

### Population and sampling

A sample of 619 schools was selected by Statistics Sweden to represent students in the three age groups. Schools were contacted by email and telephone until desired number of participants from each school grade had been included. The survey was conducted during the school year of 2016–2017, and around half of the students participated during each term. The survey was conducted class wise, and each class was visited during one day. Parents and students received written information some weeks before the visit. Students also received verbal information and instructions on the day of the survey. Students could opt out at any stage of the survey without giving any reason.

Of 5145 invited students, 3477 participated in the survey (68 %). The non-participation analyses showed that the participants were representative with regard to socio-demographic factors such as household education, income and type of municipality^(27)^. For the purpose of the current study, we included 2905 participants with complete information on food intake and household education (84 % of participating students).

### Dietary assessment

RiksmatenFlexDiet is a dietary assessment tool based on the 24-h recall method and could be completed on a computer, tablet or smart phone. Validation of the method concluded that RiksmatenFlexDiet provides information on intake of energy, fruit, vegetables and wholegrains that is at least as valid as information from 24-h recall interviews^(28)^. A food list adapted for adolescents (SFA food composition database, version Riksmaten adolescents 2016–2017) containing 778 foods, dishes and beverages was used. Participants registered their food intake during three days. The first day was completed during the school visit, and the third day was randomly assigned to occur 4–10 d later. The first and third days were retrospective, and the second day was partially prospective since it was initiated during the school visit. Data from the two retrospective days were used in the current study in order to avoid mixing retrospective and prospective methods.

Participants searched for foods in the search field in the tool and chose from a returned list of foods. They then specified the amount consumed through pieces, portion pictures or household measurements. Many of the foods were generic and were calculated based on a recipe of, for example, a meat stew. In a second step, the meat and sauce base of the stew could be specified. This made the total number of actual foods almost 2300. To facilitate the finding of the correct food, picture series containing 4–16 typical foods were available for five food groups (breakfast cereals, breads, sandwiches, fat spreads and ice creams). Automatic reminders to register beverages and condiments appeared throughout the registration. To complement the 24-h method, students filled in non-quantitative food propensity questionnaires for selected foods. Energy and nutrient intake was calculated automatically from the registered food intake.

### Development of dietary indices

The SFA issued the latest Swedish food-based dietary guidelines in 2015, summarised in the sentence ‘Find *your* way to eat greener, not too much and be active’^(29)^ and hereafter referred to as ‘Find *your* way’. The guidelines contain food-specific advice as well as recommendations about variation of the diet. The advice about variation is qualitative without specific amounts or frequencies (‘Variety is the spice of life’ and ‘Eat fish in various ways’). ‘Find *your* way’ emphasises the importance of varying food intake in order to increase the possibilities to cover nutrient requirement and decrease the risk of exposure to contaminants^(30)^.

### Swedish Healthy Eating Index for Adolescents 2015

The components of the Swedish Healthy Eating Index for Adolescents (SHEIA15) were based on the key advices of ‘Find *your* way’ and the Nordic Nutrition Recommendations from 2012^(2)^ as presented in Table 1. The calculations were adapted from Knudsen *et al.*^(32)^ and were constructed as the ratio between the actual intake and the recommended intake of each food or nutrient included in the score (Table 1). This construction takes all amounts into account and not only those that reach the cut-offs. Values below zero and above one were recoded to zero and one, respectively.

**Table 1:** Components of the Swedish Healthy Eating Index for Adolescents 2015 (SHEIA15) and the key advice behind each component

Key diet advice	Recommendation/advice	SHEIA15 component	Calculation
‘More vegetables and fruits’	At least 500 g vegetables and fruit per day* (potatoes not included) 2·5 g fibre/MJ†	Intakes of total vegetables, including root vegetables and pulses, fruits and berries expressed as g/d Intake of fibre, expressed as g/MJ	Vegetables and fruits: Intake/500 Fibre: Intake/2·5
‘Switch to wholemeal’	At least 75 g wholemeal/10 MJ‡	Intake of wholemeal, expressed as g/10 MJ	Intake/75
‘More seafood’	45 g fish and shellfish per day (frequency of 2–3 per week and portion size 125 g)§	Intakes of fish and shellfish expressed as g/d	Intake/45
‘Switch to healthy fats’ ‘Switch to low fat dairy products’	PUFA minimum 7·5 E%† MUFA minimum 15 E%† SFA maximum 10 E%†	Intakes of PUFA, MUFA and SFA expressed as E%	PUFA: E%/7·5 MUFA: E%/15 SFA: 1 − ((E% − 10)/10)
‘Less red and processed meat’	Maximum 500 g red and processed meat per week*	Intakes of red and processed meat expressed as g/week	1 − ((intake − 500)/500)
‘Less sugar’	Maximum 10 E% added sugar†	Intake of added sugar expressed as E%	1 − ((E% − 10)/10)

PUFA, Polyunsaturated Fatty Acids; MUFA, Monounsaturated fatty acids; SFA, Saturated Fatty Acids; E%, percent of total energy.

* Based on Swedish food-based dietary guidelines^(29)^.

† Based on the Nordic Nutrient Recommendations^(2)^.

‡ Based on the nutrient density as described by Becker *et al.*^(31)^.

§ Based on Swedish food-based dietary guidelines^(29)^ and personal communication (Brugård Konde).

The individual scores were added to a maximum score of 9. The score was normally distributed, and for the analyses, it was divided into quartiles with the middle two quartiles combined into one group. Quartile 1 (low group) consisted of 709 participants, quartiles 2–3 (medium group) of 1471 participants and quartile 4 (high group) of 725 participants. This division was comparable with Knudsen *et al.*^(32)^.

### Riksmaten Adolescents Diet Diversity Score

The construction of Riksmaten Adolescents Diet Diversity Score (RADDS) was based on the food groups stated in relation to the diversity in ‘Find *your* way’ and does not take amounts or frequencies into account. The food subgroups included in the score are described in Table 2.

**Table 2:** Food subgroups included in the Riksmaten Adolescents Diet Diversity Score (RADDS)

Food subgroups	Comment
Cabbage	Raw and cooked, as side dish or main dish, including vegetables from composite dishes
Root vegetables	
Pulses	
Other vegetables	
Fruit	Excluding dried fruits, juice and deserts. Including smoothies
Berries	
Wholegrain varieties of pasta, rice, grains and bread	Not including composite dishes
Red meat	As main dish or main component in composite dishes
Poultry	
Vegetarian protein (dishes with pulses, replacement products)	
Egg and egg dishes	Not including egg from composite dishes
White fish	As main dish or main component in composite dishes
Oily fish	
Shellfish	
Fermented dairy products	Not including composite dishes
Milk	
Milk replacements	

It is possible to disaggregate composite dishes that are registered in RiksmatenFlexDiet. If a participant registered fish soup, the vegetables and other ingredients included in that dish can be extracted from the database. However, in the composite dishes containing pasta, rice or other grains, participants could not indicate whether it was a wholegrain variety or not. Thus, only consumption of specific wholegrain products could be included (Table 2). No point was given for consumption of foods such as deserts with fruit/berries, juice and dried fruit, since these foods are not included in the dietary guidelines.

Participants were given one point for consumption above 5 g from each subgroup. The maximum point was 17, but no participant reached more than 13 points, resulting in a distribution skewed towards lower scores. As for SHEIA15, we created groups with higher and lower scores by dividing the score in three groups. Roughly, one quarter of participants were allocated to the lowest scores (<4 points, *n* 734) and one quarter to the highest scores (>7 points, *n* 521). The remaining participants were in the middle group (4–7 points, *n* 1650).

### Socio-demographic factors

The socio-demographic factors used were sex and age, both collected from the class lists, along with household education and school municipality. The parents answered questions about their own and their partner’s highest degree of education in the web-based tool. Five levels of education were classified into ≤12 years and >12 years of education. Each school municipality was allocated to one of the three types of municipalities according to the classification by Swedish Association of Local Authorities and Regions^(33)^. The classification is based on structural parameters such as population and commuting patterns and is divided into ‘Large cities and municipalities near large cities’ (labelled Large cities); ‘Medium-sized towns and municipalities near medium-sized towns’ (labelled Medium-sized towns) and ‘Smaller towns/urban areas and rural municipalities’ (labelled Smaller towns).

### Anthropometry

Weight and height were measured by field staff using standardised methods and portable equipment. Weight was measured to the nearest 0·1 kg using SECA 862 or 899 digital weighing scales. Height was measured to the nearest 0·1 cm using SECA 213 portable stadiometers. BMI was calculated (kg/m^2^), and the International Obesity Task Force reference, taking sex and age into account, was used to determine weight status^(34)^.

## Evaluation of energy intake

Since misreporting is a well-known bias in diet assessment, an evaluation of the energy intake was performed. The energy intake was compared with the total energy expenditure calculated according to the methods of Goldberg and Black^(35,36)^ using information from accelerometers, body weight and height. As described by Lemming *et al.*^(10)^, each participant was classified into under-, plausible- or over-reporter of energy.

## Statistics

Comparison of the distribution of participants and non-participants according to school grade and sex was analysed with Pearson’s *χ*^2^.

Difference of proportions of high, medium and low scores of SHEIA15 and RADDS across groups was determined by evaluating the CI. Overlapping CI indicate no statistical difference between groups. Pearson’s correlation was used to assess the relationship between SHEIA15 and RADDS. Difference in mean scores on RADDS between omnivores and vegetarians/other food preferences was analysed with *t* test.

The consumption and nutrient data in the current study were transformed from current intake to habitual intake using the statistical method, Multiple Source Method^(37,38)^. The transformation was stratified by school grade since energy intake differed between the groups^(9)^. In the Multiple Source Method model, all participants were assumed to be consumers of all foods except fish where frequency of consumption was collected from the food propensity questionnaires. Only 50 % had consumed fish during the two registration days but 90 % stated that they generally eat fish. The food propensity questionnaire was thus used to identify true non-consumers.

Intakes of macro- and micronutrients were normally distributed, and these intakes were compared between high, medium and low scores of SHEIA15 and RADDS using one-way ANOVA with Bonferroni corrections. Intake of food groups had non-normal distributions, and differences between groups were analysed with Kruskal–Wallis rank test and Dunn’s multiple-comparison test with Bonferroni corrections.

The two indices were then analysed as continuous dependent variables in two separate multilevel mixed-effects linear regression analyses, mutually adjusting for household education, sex, school grade, weight status and school municipality. Schools were included as random effect to allow students to cluster within schools. Intra-class correlations were retrieved with the *estat ICC* command in Stata. Sensitivity analyses were performed including only plausible energy reporters in the multilevel mixed-effects linear regression analyses. For SHEIA15, the score was calculated based on dietary intake as mean of 2 d in addition to the Multiple Source method and included in sensitivity analysis.

All analyses were conducted in Stata version 14.2, StataCorp.

## Results

Mean age of participants was 12 years in school grade 5, 15 years in school grade 8 and 18 years in school grade 11 (Table 3). Included participants were distributed equally among school grades (33 % in each grade). In contrast, the excluded participants without complete data on diet and parental education (*n* 572) were more likely to attend school grade 8, 41 %, and less likely to attend school grade 11, 23 % (*P* < 0·001). Excluded participants were also more likely to be boys, 62 % compared with 44 % among included participants (*P* < 0·001).

**Table 3:** Characteristics of participants by school grade and sex

	School grade 5				School grade 8				School grade 11			
	Boys (*n* 451)		Girls (*n* 538)		Boys (*n* 429)		Girls (*n* 535)		Boys (*n* 380)		Girls (*n* 552)	
	Mean	sd	Mean	sd	Mean	sd	Mean	sd	Mean	sd	Mean	sd
Age (years)	11·5	0·4	11·5	0·4	14·5	0·4	14·5	0·4	17·7	0·6	17·7	0·6
SHEIA15 (mean score)	5·7	0·7	5·9	0·7	5·7	0·8	6·0	0·8	5·5	0·9	6·0	0·9
RADDS (mean score)	5·6	1·7	6·1	1·7	5·6	2·0	6·1	2·0	5·4	1·9	5·9	2·0

	%	95 % CI	%	95 % CI	%	95 % CI	%	95 % CI	%	95 % CI	%	95 % CI
Overweight including obesity	22·0	18·0, 26·0	22·0	19·0, 26·0	17·0	14·0, 21·0	17·0	14·0, 20·0	26·0	22·0, 31·0	22·0	18·0, 25·0
Household education												
≤12	39·0	35·0, 44·0	35·0	31·0, 39·0	39·0	34·0, 43·0	35·0	31·0, 39·0	42·0	37·0, 47·0	44·0	40·0, 48·0
>12	61·0	56·0, 65·0	65·0	61·0, 69·0	61·0	57·0, 66·0	65·0	61·0, 69·0	58·0	53·0, 63·0	56·0	52·0, 60·0
School municipality												
Large cities	31·0	27·0, 35·0	36·0	32·0, 41·0	28·0	24·0, 32·0	26·0	22·0, 30·0	29·0	24·0, 33·0	24·0	20·0, 27·0
Medium-sized towns	40·0	36·0, 44·0	32·0	28·0, 36·0	53·0	48·0, 57·0	57·0	52·0, 61·0	38·0	34·0, 43·0	36·0	32·0, 40·0
Smaller towns	29·0	25·0, 33·0	32·0	28·0, 36·0	20·0	16·0, 24·0	17·0	14·0, 21·0	33·0	28·0, 38·0	40·0	36·0, 45·0
Energy reporters												
Plausible reporters	59·0	54·0, 63·0	63·0	58·0, 67·0	56·0	51·0, 60·0	59·0	55·0, 64·0	58·0	53·0, 63·0	61·0	57·0, 65·0

SHEIA15, Swedish Healthy Eating Index for Adolescents 2015; RADDS, Riksmaten Adolescents Diet Diversity Score.

Mean scores of SHEIA15 and RADDS were slightly higher in girls in all age groups. There were no differences in prevalence of overweight between girls and boys in any school grade. Proportion of household education was similar across sexes and school grades, with a higher proportion of household education >12 years. Distribution of school municipalities was similar between girls and boys within the same school grade but varied across school grade: in grade 8, more than half of students attended schools in medium-sized cities, while the proportion was around 30–40 % in grade 5 and 11. Between 56 and 63 % of all participants were plausible energy reporters (Table 3).

### SHEIA15 and RADDS in relation to socio-demographic factors

A low SHEIA15 score was more common for boys, the school grade 11, participants with low household education and a low RADDS score (Table 4). A high SHEIA15 score was more common for girls, students with high household education, high RADDS score as well as participants in school grade 8 compared with school grade 5.

**Table 4:** Mean score of Swedish Healthy Eating Index for Adolescents 2015 (SHEIA15) and Riksmaten Adolescents Diet Diversity Score (RADDS) across index groups. Proportion of index groups according to socio-demographic factors and weight status, percentages and 95 % CI

	SHEIA15 group						RADDS group					
	Low (*n* 709)		Medium (*n* 1471)		High (*n* 725)		Low (*n* 734)		Medium (*n* 1650)		High (*n* 521)	
%	25		50		25		25		57		18	
	Mean	sd	Mean	sd	Mean	sd	Mean	sd	Mean	sd	Mean	sd
SHEIA15 mean	4·8	0·4	5·8	0·3	6·9	0·4	5·3	0·8	5·9	0·7	6·4	0·8
RADDS mean	4·6	1·6	5·9	1·6	7·0	1·9	3·5	0·8	6·0	0·8	8·7	0·9

	%	95 % CI	%	95 % CI	%	95 % CI	%	95 % CI	%	95 % CI	%	95 % CI
Sex												
Girls	19·0	17·0, 21·0	51·0	49·0, 54·0	30·0	27·0, 32·0	21·0	19·0, 23·0	58·0	55·0, 60·0	21·0	19·0, 23·0
Boys	31·0	28·0, 34·0	50·0	47·0, 53·0	19·0	17·0, 21·0	30·0	28·0, 33·0	56·0	53·0, 59·0	14·0	12·0, 16·0
School grade												
Grade 5	20·0	18·0, 23·0	59·0	56·0, 62·0	21·0	19·0, 24·0	22·0	20·0, 25·0	60·0	57·0, 63·0	18·0	15·0, 20·0
Grade 8	24·0	22·0, 27·0	47·0	44·0, 51·0	28·0	25·0, 31·0	27·0	24·0, 30·0	54·0	50·0, 57·0	20·0	17·0, 22·0
Grade 11	29·0	26·0, 32·0	45·0	42·0, 48·0	26·0	23·0, 29·0	27·0	24·0, 30·0	57·0	53·0, 60·0	16·0	14·0, 19·0
Weight status												
UW/NW	24·0	22·0, 26·0	51·0	49·0, 53·0	25·0	23·0, 27·0	24·0	22·0, 25·0	58·0	56·0, 60·0	19·0	17·0, 20·0
OW/OB	27·0	23·0, 31·0	49·0	45·0, 53·0	24·0	21·0, 28·0	32·0	29·0, 36·0	53·0	49·0, 57·0	15·0	12·0, 18·0
Household education												
≤12	30·0	27·0, 33·0	49·0	47·0, 52·0	21·0	18·0, 23·0	32·0	29·0, 35·0	55·0	52·0, 58·0	13·0	11·0, 15·0
>12	21·0	19·0, 23·0	51·0	49·0, 54·0	28·0	26·0, 30·0	21·0	19·0, 23·0	58·0	55·0, 60·0	21·0	19·0, 23·0
School municipality												
Larger cities	22·0	19·0, 24·0	51·0	47·0, 54·0	28·0	25·0, 31·0	26·0	24·0, 30·0	56·0		18·0	15·0, 20·0
Medium cities	25·0	23·0, 28·0	50·0	47·0, 53·0	25·0	23·0, 28·0	25·0	23·0, 28·0	56·0		19·0	17·0, 21·0
Rural areas/towns	26·0	23·0, 29·0	52·0	48·0, 55·0	22·0	19·0, 25·0	24·0	22·0, 27·0	58·0		17·0	15·0, 20·0
RADDS group												
Low	50·0	46·0, 53·0	42·0	39·0, 46·0	8·0	7·0, 11·0	–		–		–	
Medium	19·0	17·0, 21·0	57·0	54·0, 59·0	24·0	22·0, 27·0	–		–		–	
High	6·0	4·0, 8·0	44·0	40·0, 48·0	50·0	46·0, 54·0	–		–		–	
SHEIA15 group												
Low	–		–		–		51·0	48·0, 55·0	44·0	41·0, 48·0	4·0	3·0, 6·0
Medium	–		–		–		21·0	19·0, 23·0	63·0	61·0, 66·0	16·0	14·0, 18·0
High	–		–		–		8·0	7·0, 11·0	56·0	52·0, 59·0	36·0	33·0, 40·0

UN/NW, underweight/normal weight; OW/OB, overweight/obese.

A low RADDS was more common among boys, overweight students, those with low household education and in those with low SHEIA15 scores (Table 4). A high RADDS score was more common among girls, those with high household education and high SHEIA15 score. Medium RADDS was similar across most groups.

When comparing the ranking of individuals in the categories of SHEIA15 and RADDS, only 3 % of participants were in opposite categories, while 43 % were in adjacent categories and 54 % were in the same categories. Pearson’s correlation showed a positive correlation between SHEIA15 and RADDS (*r* = 0·5, *P* < 0·001).

### SHEIA15 and RADDS in relation to dietary intakes

#### SHEIA15

Mean score and intake for each component of SHEIA15 are presented in Table 5. Food and nutrient intakes across SHEIA15 groups are presented in Table 6. Energy intake was higher in the group with low SHEIA15 scores compared with high scores. Energy-adjusted intake of fibre, wholegrains, PUFA and *n*-3 fatty acids was higher, while intake of energy-adjusted added sugar and saturated fat was lower in the high SHEIA15 group. Further, for all vitamins and minerals, the energy-adjusted intake increased with higher scores. Consumption of sugar-sweetened beverages, sweets and red and processed meat was lower in the high score group, while juice, vegetables and fruits and fish intakes were higher. For dairy products, consumption was higher in the middle group comparing both lower and higher scores.

**Table 5:** Mean scores and mean intake of each subcomponent of SHEA15

	SHEIA15 score		Mean intake	
	Mean	sd	Gram	sd
Vegetables and fruit	0·5	0·2	232·0	102·9
Fibre	0·8	0·2	35·6	21·1
Fish and shellfish	0·5	0·3	22·9	14·5
Red and processed meat	0·6	0·3	97·4	35·2

	Mean	sd	E%	sd
SFA	0·6	0·2	13·7	2·0
Mono-SFA	0·9	0·1	13·6	2·1
Poly-SFA	0·6	0·1	4·7	0·9
Added sugar	0·8	0·2	10·5	3·5

	Mean	sd	Gram/MJ	sd
Wholegrains	0·5	0·2	2·1	0·5

SHEIA15, Swedish Healthy Eating Index for Adolescents 2015.

**Table 6:** Food and nutrient intakes according to Swedish Healthy Eating Index for Adolescents 2015 (SHEIA15)

	SHEIA15 group					
	Low (*n* 709)		Medium (*n* 1471)		High (*n* 735)	
Nutrient intake*	Mean	sd	Mean	sd	Mean	sd
Energy (MJ)^a^	9·6	2·6	8·8	2·4	8·4	2·2
Fibre (g/MJ)^a^	1·7	0·3	2·0	0·4	2·6	0·6
Wholegrains (g/10 MJ)^a^	23·2	12·1	33·9	17·7	50·9	24·9
Added sugar (E%)^a^	12·3	4·3	10·4	3·1	9·0	2·6
Saturated fat (E%)^a^	14·6	2·1	13·8	1·9	12·8	1·9
Monounsaturated fat (E%)^d^	13·6	2·1	13·6	2·1	13·5	2·1
Polyunsaturated fat (E%)^a^	4·3	0·7	4·6	0·8	5·2	1·0
*n*-3 fatty acids (E%)^a^	0·8	0·2	0·9	0·2	1·0	0·2
Intake of vitamins and minerals*						
Vitamin D (µg/10 MJ)^a^	5·9	2·2	6·8	2·4	7·2	2·6
Vitamin C (mg/10 MJ)^a^	70·0	32·0	89·0	41·0	108·0	43·0
Folate (µg/10 MJ)^a^	256·0	58·0	296·0	58·0	347·0	76·0
Fe (mg/10 MJ)^a^	8·9	1·6	9·4	1·6	10·3	1·7
Iodine (µg/10 MJ)^a^	250·0	65·0	279·0	69·0	315·0	138·0
Se (µg/10 MJ)^a^	43·0	11·0	49·0	14·0	54·0	18·0
Food intake (g/10 MJ)†	Median	p25–p75	Median	p25–p75	Median	p25–p75
Sugar-sweetened beverages^a^	243·0	148·0–366·0	197·0	131·0–279·0	164·0	107·0–234·0
Juice^b^	17·0	12·0–137·0	21·0	13·0–162·0	22·0	13·0–162·0
Sweets^a^	31·0	13·0–57·0	25·0	14·0–45·0	23·0	13·0–40·0
Red and processed meat^a^	127·0	104·0–156·0	109·0	89·0–134·0	90·0	70·0–116·0
Vegetables and fruits^a^	183·0	139·0–238·0	250·0	194·0–316·0	346·0	278·0–430·0
Fish and shellfish^a^	15·0	10·0–23·0	24·0	15·0–36·0	36·0	23·0–49·0
Dairy products^c^	292·0	151·0–520·0	361·0	182·0–553·0	294·0	155·0–510·0

^a^Significant differences between all groups; ^b^Low SHEIA15 group significantly different from medium and high SHEIA15 groups; ^c^Middle SHEIA15 group significantly different from low and high SHEIA15 groups; ^d^No significant difference.

* One-way ANOVA with Bonferroni corrections.

† Kruskal–Wallis rank test and Dunn’s multiple-comparison test with Bonferroni corrections.

**Table 7:** Mean scores and mean intake of each subcomponent of RADDS

	RADDS score		Mean intake	
	Mean	sd	Gram	sd
Cabbage	0·1	0·3	6·4	22·6
Root vegetables	0·5	0·5	16·1	29·7
Pulses	0·1	0·3	3·8	18·1
Other vegetables	1·0	0·2	115·8	98·6
Fruit	0·5	0·5	68·9	102·3
Berries	0·2	0·4	7·0	23·0
Wholegrain varieties of pasta, rice, grains and bread	0·5	0·5	82·8	97·4
Red meat	0·8	0·4	52·6	57·9
Poultry	0·4	0·5	29·1	51·7
Vegetarian protein (dishes with pulses, replacement products)	0·2	0·4	9·4	30·3
Egg and egg dishes	0·2	0·4	11·3	34·7
White fish	0·2	0·4	9·3	27·6
Oily fish	0·2	0·4	9·8	29·0
Shellfish	0·1	0·2	2·3	12·6
Fermented dairy products	0·3	0·5	56·8	101·2
Milk	0·7	0·5	279·2	333·6
Milk replacements	0·0	0·2	6·9	58·4

RADDS, Riksmaten Adolescents Diet Diversity Score.

#### RADDS

Mean score and intake of each subgroup of RADDS are described in Table 7. Food and nutrient intakes across RADDS groups are presented in Table 8. Intake of energy as well as energy-adjusted intake of fibre, wholegrains, PUFA and *n*-3 fatty acids increased with higher RADDS. On the contrary, intake (E%) of added sugar, MUFA and SFA decreased. Energy-adjusted intake of all vitamins and minerals was higher in the high RADDS group compared with those with low RADDS. Consumption (g/10 MJ) of sugar-sweetened beverages and red and processed meat was lower in the high RADDS group, while consumption of vegetables and fruits, fish and dairy products was higher. Consumption (g/10 MJ) of juice and sweets was similar across groups. Two hundred and nine participants reported that they were vegetarians, vegans or had other food preferences, possibly affecting their opportunity to score high on RADDS. However, there was no difference in mean score between groups (5·8 in the omnivore group and 5·9 in the vegetarian/other group (*P* = 0·56).

**Table 8:** Food and nutrient intake according to Riksmaten Adolescents Diet Diversity Score (RADDS)

	RADDS group					
	Low (*n* 734)		Medium (*n* 1650)		High (*n* 521)	
Nutrient intake*	Mean	sd	Mean	sd	Mean	sd
Energy (MJ)^a^	8·5	2·5	8·9	2·4	9·3	2·2
Fibre (g/MJ)^a^	1·8	0·5	2·1	0·5	2·3	0·6
Wholegrains (g/10 MJ)^a^	28·3	19·5	36·7	20·9	42·3	21·0
Added sugar (E%)^b^	11·4	4·2	10·3	3·3	9·9	3·0
Saturated fat (E%)^a^	14·0	2·2	13·7	2·0	13·4	1·9
Monounsaturated fat (E%)^b^	13·9	2·3	13·5	2·0	13·3	2·0
Polyunsaturated fat (E%)^a^	4·6	0·9	4·7	0·9	4·8	0·9
*n*-3 fatty acids (E%)^a^	0·9	0·2	0·9	0·2	1·0	0·2
Intake of vitamins and minerals*						
Vitamin D (µg)^a^	5·9	2·5	6·8	2·4	7·6	2·4
Vitamin C (mg)^a^	75·0	44·0	91·0	40·0	103·0	38·0
Folate (µg)^a^	260·0	63·0	303·0	64·0	341·0	72·0
Fe (mg)^a^	9·0	1·6	9·6	1·7	10·1	1·7
Iodine (µg)^a^	252·0	69·0	288·0	106·0	300·0	69·0
Se (µg)^a^	44·0	13·0	49·0	15·0	54·0	16·0
Food intake (g/10 MJ)†	Median	p25–p75	Median	p25–p75	Median	p25–p75
Sugar-sweetened beverages^a^	252·0	165·0–372·0	184·0	123·0–269·0	163·0	105·0–243·0
Juice^c^	20·0	13·0–145·0	20·0	13·0–162·0	20·0	12·0–153·0
Sweets^c^	27·0	14·0–50·0	25·0	13·0–46·0	25·0	13·0–45·0
Red and processed meat^a^	120·0	95·0–149·0	109·0	87·0–134·0	95·0	73·0–121·0
Vegetables and fruits^a^	195·0	144·0–262·0	260·0	195·0–335·0	308·0	252·0–391·0
Fish and shellfish^a^	19·0	12·0–32·0	24·0	15·0–38·0	29·0	18·0–41·0
Dairy products^a^	227·0	120·0–448·0	359·0	184·0–554·0	382·0	223·0–580·0

^a^Significant differences between all groups; ^b^Low RADDS group significantly different from medium and high RADDS groups; ^c^No significant difference.

* One-way ANOVA with Bonferroni corrections.

† Kruskal–Wallis rank test and Dunn’s multiple-comparison test with Bonferroni corrections.

### Multilevel mixed-effects linear regression analyses

In the multilevel analyses including SHEIA15 as a continuous variable, girls and adolescents from households with higher education had significantly higher scores (Table 9). School grade, weight status and school municipality were not associated with SHEIA15.

**Table 9:** Associations of Swedish Healthy Eating Index for Adolescents 2015 (SHEIA15) and Riksmaten Adolescents Diet Diversity Score (RADDS) with socio-demographic factors and weight status*

	SHEIA15					RADDS				
	Mean	sd	Coefficient	95 % CI	*P*-value	Mean	sd	Coefficient	95 % CI	*P*-value
Intercept			5·50	5·32, 5·69	<0·001			4·90	4·49, 5·30	<0·001
Sex										
Boys	5·6	0·8				5·6	1·8			
Girls	5·9	0·8	0·28	0·22, 0·34	<0·001	6·0	1·9	0·48	0·34, 0·61	<0·001
School grade										
Grade 5	5·8	0·7				5·9	1·7			
Grade 8	5·8	0·8	0·05	−0·07, 0·17	0·42	5·9	2·0	−0·003	−0·25, 0·25	0·98
Grade 11	5·8	0·9	−0·03	−0·16, 0·09	0·61	5·7	2·0	−0·13	−0·39, 0·13	0·32
Weight status										
UW/NW	5·8	0·8				5·9	1·9			
OW/OB	5·8	0·8	−0·01	−0·08, 0·06	0·84	5·5	1·9	−0·29	−0·45, −0·12	0·001
Household education										
<12	5·7	0·8				5·4	1·8			
>12	5·9	0·8	0·13	0·07, 0·19	<0·001	6·1	1·9	0·56	0·42, 0·70	<0·001
School municipality										
Larger cities	5·9	0·8				5·8	1·9			
Medium cities	5·8	0·8	−0·08	−0·21, 0·05	0·23	5·9	1·9	0·18	−0·09, 0·45	0·18
Rural areas/towns	5·8	0·8	−0·13	−0·27, 0·01	0·07	5·8	1·8	0·14	−0·15, 0·43	0·42

UN/NW, underweight/normal weight; OW/OB, overweight/obese.

* SHEIA15 and RADDS as continuous dependent variables in two separate multilevel mixed-effects linear regression analyses, mutually adjusting for household education, sex, school grade and weight status and school municipality. Schools were included as random effect to allow students to cluster within schools.

In the multilevel analyses including RADDS as a continuous variable, girls, normal-weight adolescents and adolescents from households with higher education had significantly higher scores (Table 9). School grade and school municipality were not associated with RADDS.

For SHEIA15, an intra-class correlation of 0·095 indicates that almost 10 % of the total variation of the score was explained by children attending the same schools. In RADDS, the corresponding number was around 7 % (intra-class correlation = 0·065).

### Sensitivity analyses

Including only plausible energy reporters did not significantly alter the results in the multilevel analyses with either SHEIA15 or RADDS (data not shown). In addition to using habitual intake when calculating SHEIA15, the mean intake of 2 d was also used. The results of the analyses only changed marginally when mean intake was used instead (data not shown).

## Discussion

The current study describes the construction of two new indices: SHEIA15 based on the Swedish food-based dietary guidelines from 2015 and RADDS that captures diet diversity. Both indices were associated with higher diet quality, that is higher intake of vegetables and wholegrains and lower intake of added sugars and red and processed meat. Boys and participants in households with lower education level scored lower on both indices.

Adolescents with higher SHEIA15 had higher energy-adjusted intakes of fibre, wholegrains, PUFA and *n*-3 fatty acids but lower intakes of energy, added sugar and SFA. Adolescents with high SHEIA15 also had higher energy-adjusted intakes of selected key nutrients that are not included in the calculation of SHEIA15. Further, food intake was more favourable^(1)^ in those with high SHEIA15; they had higher intakes of fish, fruits and vegetables but lower intakes of red meat and sweets. This shows that SHEIA15 captures diet quality in a holistic way and is thus useful in analyses where it is an advantage to capture overall diet quality. This is similar to other scores, for example, those based on American^(39)^ and Finnish^(13)^ dietary guidelines, which also found positive associations with high diet quality. Comparing points on the different scores is not possible since they include somewhat different components and calculations but results indicate that the overall associations are comparable. There are a large number of other diet quality scores, but results cannot be compared since the constructions of the scores differ radically^(40–43)^.

Similar to SHEIA15, those with high scores on RADDS had higher diet quality. Energy-adjusted intake of nutrients and food groups shows similar patterns between the two indices. Our results confirm results from other studies showing that variety is associated with better diet quality. Ramsay *et al.* found that variety of fruits and vegetables was linked to overall diet quality in US children^(44)^. Diet diversity was also associated with diet quality in American adults using the US Healthy Food Diversity index^(45)^. Further, in a subsample of The National Health and Nutrition Examination Survey, the US Healthy Food Diversity index was inversely associated with obesity indicators but positively associated with energy intake in both sexes^(46)^. Similarly, we found that a larger variation of healthy foods was associated with higher energy intake as well as a lower proportion of overweight/obesity. This is in contrast to SHEIA15, where no differences between weight status groups were observed and energy intake decreased across groups. This indicates that RADDS captures additional dimensions of dietary habits. RADDS may be less biased and thus less prone to misreporting since it is documented that overweight/obese individuals often misreport to a larger extent than normal-weight individuals^(47)^. In contrast, SHEIA15 captures societal norms of healthy diets and might thus be more prone to misreporting.

Multivariate adjusted results on both indices showed that girls scored higher than boys and adolescents in households with high education scored higher than adolescents in households with low education. This is in line with results from other studies. A Finnish study found that low maternal education was associated with low scores on the Finnish Children Healthy Eating Index among children aged 1–6 years^(13)^. In older adults, in the British EPIC cohort, lower social class was associated with lower fruit and vegetable variety^(24)^. It is worrying that these differences persist even though they are well known^(8)^. Likewise, it is known that girls and women eat healthier than men, and sex differences occur in both adults^(48)^ and adolescents^(9,10)^ in Sweden. Our findings suggest that the differences in food habits are established already at a young age. Since unhealthy dietary habits are associated with the long-term development of non-communicable diseases, such as CVD and type 2 diabetes^(1)^, the results are alarming. Dietary habits are modifiable risk factors that have major public health implications, and improving them should be a priority. Promoting a varied diet could be an effective tool to improve the overall diet since both scores were equally associated with a healthy food and nutrient intake.

We did not find any associations between school municipality and either score. In a previous report on the food intake of this population, intake of individual foods did not differ between areas^(9)^ and thus it might be expected that the composite scores would not differ either. The fact that other studies have found conflicting results between area and dietary habits could imply that the definition of urban and rural areas differs across countries. In addition, different levels of information can be used, residential area or school municipality as in our study.

Diet diversity and healthy diet scores are often based on data from frequency questionnaires instead of registered food intake^(23,24,49,50)^. Our detailed data allow us to utilise information on amounts and types of foods and to include ingredients from composite dishes. To account for the day-to-day variation within participants that occur with the 24-h recall method, the data were converted to habitual intake using the Multiple Source Method^(37)^. Further strengths of the current study are the objective and standardised measurements of height and weight as well as physical activity that enables sensitivity analyses using plausible energy reporters only.

A limitation of the current study is that we lack information on other individual and area level factors such as income, occupational position or area deprivation. All these factors may influence dietary habits and might confound the associations found in the current study. Healthy eating was associated with higher costs in a Swedish study^(51)^ which found the cheapest and most unhealthy diets among children with parents with low education and manual low-skill occupations. We do not know if the association we found with education is independent of income and other socio-demographic factors. Further research on the multifactorial, socio-demographic influence on diet quality and diversity in Swedish adolescents is desirable.

## Conclusions

The new indices on healthy eating and diet diversity can be used to capture the diet in a holistic way in adolescents. They can identify risk groups as done in this population where less healthy and less varied diets were found in boys and adolescents in households with lower education. The variety score was associated with overweight and obesity, while the healthy eating index was not. Both scores were associated with healthy dietary habits, indicating that encouraging a varied diet can be a tool in public health promotion.
